# Enzymatic Synthesis and Characterization of a Novel α-1→6-Glucosyl Rebaudioside C Derivative Sweetener

**DOI:** 10.3390/biom9010027

**Published:** 2019-01-14

**Authors:** Zheng Yang, Brandon Uhler, Ted Zheng, Kristie M. Adams

**Affiliations:** 1 Cargill Inc., 14800 28th Ave N, Plymouth, MN 55447, USA; buhler8819@gmail.com; 2 Cargill Inc., 3201 Needmore Rd, Dayton, OH 45414, USA; ted_zheng@cargill.com; 3 Steelyard Analytics, Inc., 704 Quince Orchard Rd., Ste. 130, Gaithersburg, MD 20878, USA; kristie.adams@steelyardanalytics.com

**Keywords:** steviol glycosides, diterpene glycosides, high intensity sweetener, enzyme modified stevia, glycosylated steviol glycosides, rebaudioside C, characterization, α-1→6-glucosyl rebaudioside C

## Abstract

Zero-calorie high-intensity sweeteners from natural sources perform very well in the market place. This has encouraged food scientists to continue the effort to search for novel natural ingredients to satisfy consumer demand. Rebaudioside C (reb C) is the third most prevalent steviol glycoside in the leaves of the *Stevia rebaudiana* Bertoni plant, but has limited applications in food and beverage products due to its low sweetness and high lingering bitterness compared to other major steviol glycosides, such as rebaudioside A (reb A). Here we present a new enzyme modification strategy to improve the taste profile of reb C by using Cargill’s propriety enzyme and sucrose as a glucose donor. A novel α-1→6-glucosyl reb C derivative was produced and its structure was elucidated by mass spectrometry and NMR spectroscopy. Sensory analysis demonstrated that this new reb C derivative has improved sweetness, reduced bitterness, and enhanced solubility in water.

## 1. Introduction

U.S. retail sales of stevia sweeteners grew 11.9% in the 52-week period between August 2017 and August 2018. On the other hand, sales of high-intensity artificial sweeteners continue to slide, dropping more than 5%, according to data from Nielsen [1]. While the majority of the products are based on the major steviol glycoside component from stevia leaves, rebaudioside A (reb A), efforts have been made to work with new steviol glycosides with a better taste profile than reb A, due to its strong and unpleasant aftertaste. Those new steviol glycosides include rebaudioside D (reb D) and rebaudioside M (reb M) [2]. However, reb D and reb M appear in very low abundance in stevia leaves, making them much less accessible and more expensive. As a result, people are looking into alternative approaches to produce steviol glycosides to make them more affordable. Some of the methods being used to accomplish this include microbial fermentation [3], bioconversion [4,5], and enzyme-modified/glycosylated steviol glycoside (EMS) [6]. Although all of these approaches rely on the use of an enzyme, they are all, in fact, different. Fermentation and bioconversion aim to produce steviol glycosides that exist in nature, but with low abundance, such as the previously mentioned reb D and reb M. EMS modifies the steviol glycosides in a different way by adding the glucose moiety into the steviol glycoside backbone in a random fashion, and the end products have not been reported to be found in nature. 

**Figure 1:**
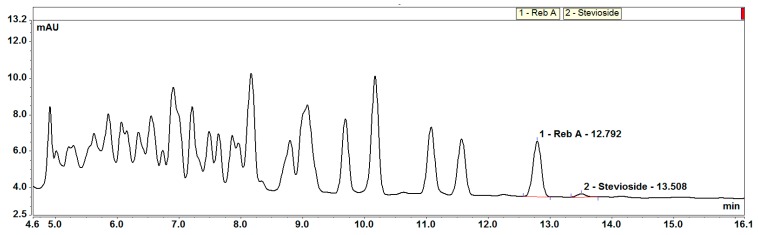
LC chromatogram of a commercial enzyme-modified/glycosylated steviol glycoside (EMS) product. Reb A: rebaudioside A.

EMS aims to reduce the cost of the products and increase their solubility. Studies have shown that EMS has an equal to better taste profile compared to its major predecessors: rebaudioside A and stevioside [7,8]. Recently, we have focused our efforts on finding new steviol glycosides with taste profiles similar to sugar, producing better steviol glycoside molecules through fermentation [9], seeking alternative natural resources for steviol glycosides [10], and using enzymes to modify the taste profile and solubility of steviol glycosides. In today’s market, all of the EMS is produced by using steviol glycosides as starting material (mainly reb A and stevioside), and maltodextrin and cyclodextrin glycosyltransferase (CGTase), α- and β-glucosidase, α- and β-galactosidase, β-fructosidase, or β-glycosyltransferase as the glycosylation agents [8]. This approach results in products of glycosylated steviol glycosides with up to 20 added glucose moieties that are difficult to characterize (Cargill data, Figure 1) [11]. An improved process has been developed that has better control over the number of glucose units added to the steviol glycoside backbone, resulting in a product that can be better characterized.

**Figure 2:**
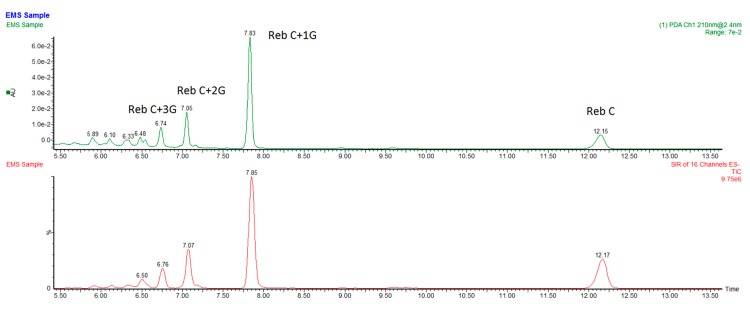
LC chromatogram of the multi-α-glycosylated reb C product. Top: LC-UV; bottom: LC-MS. Reb C: rebaudioside C. G: glucose.

**Figure 3:**
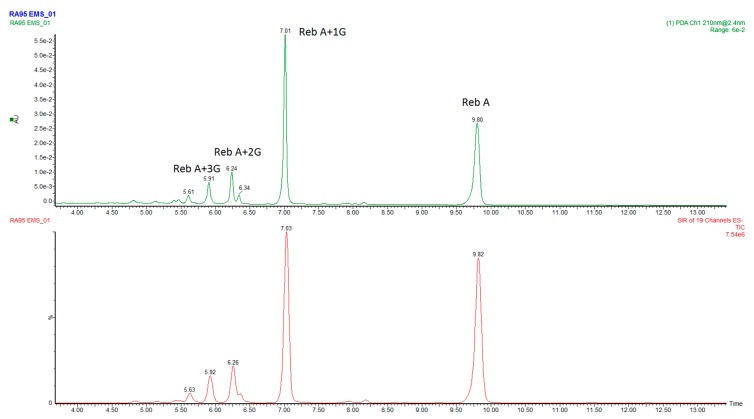
LC chromatogram of the multi-α-glycosylated reb A product. Top: LC-UV; bottom: LC-MS.

Rebaudioside C (reb C) is the third most abundant steviol glycoside in the leaves of the *Stevia rebaudiana* Bertoni plant, but has limited applications in food and beverage market due to its low sweetness and high lingering bitterness. It also has low solubility in water. Enzymatic glucosylation is an applicable approach to improve its taste profile and physical characteristics. By using rebaudioside C (reb C) and sucrose as the starting material, with Cargill’s proprietary alternansucrase derived from *Leuconostoc citreum* [12], we produced a new EMS product that has a limited number of glycosylated reb C products (Figure 2). We characterized the major component of the products, and found that it is a novel α-1→6-glycosyl rebaudioside C compound. Sensory evaluation revealed that the α-1→6 glycosylated reb C compound had improved sweetness and reduced bitterness in comparison with the parent reb C sweetener. It is our understanding that all of the α-glucosylated steviol glycoside products in the marketplace are reported as stereo-and regiospecific glycosylation via 1→4 linkage [13,14], and the 1→6 linkage occurs very rarely in the steviol glycoside family. A recent publication revealed formation of an α-1→6 linkage of a glucose addition to reb A by using glucansucrase (mutant) enzymes from *Lactobacillus reuteri* 180. This α-1→6 glycosylated reb A compound was reported to have a superior taste profile, with significantly less bitterness than the parent reb A. In fact, the author claimed that it displayed almost no bitterness at all [15]. Reb A was also modified by using the method previously described for reb C, and the product is highly likely to contain the same α-1→6 glycosylated reb A, although it has not been fully characterized (Figure 3). This new kind of α-1→6 glycosylated steviol glycoside product demonstrates improved and desirable sensory properties and can be used as a low-cost alternative to highly expensive reb D and reb M sweeteners.

## 2. Materials and Methods

### 2.1. Experimental Sections

#### 2.1.1. Enzyme Modification Process

Preliminary experiments were carried out to optimize the enzyme modification process by using different starting steviol glycoside (SG): sucrose ratios, varying enzyme dosages and reaction time. Briefly, a Cargill commercial sample of high purity reb C (>95% purity) was prepared at 0.25% *w/v* with 0.05 M citric acid buffer at pH 5.5. Sucrose (Sigma reagent grade) was prepared to 1 M with 0.05 M citric acid buffer of pH 5.5. For enzymatic reactions, 1.00 mL of the 0.25% SG substrate was first pipetted in to 2-mL vials with screw caps, followed by adding the appropriate amount of sucrose solution to form various SG: sucrose ratios on a molar basis and finally adding various amounts of alternansucrase. Two controls were included in the experiment. Control-1 consisted of SG and sucrose with no alternansucrase and control-2 consisted of SG and alternansucrase with no sucrose. The enzymatic reaction was carried out by gently inverting the vials at 55 °C up to 48 h. Aliquots of samples were taken after certain time of reaction for LC-UV and LC-MS analyses to find the best incubation conditions. Both controls at either the highest enzyme dosage or the highest SG: sucrose ratios showed identical chromatograms as the starting SG solution in LC-UV analysis (data omitted), indicating no reaction between sucrose and SG substrates nor the alternansucrase enzyme caused any change to reb C. To generate the enzyme-modified reb C product, 4.5 g sucrose was added to 500 mL of 0.25% *w/v* reb C prepared with 0.05 M citric acid buffer of pH 5.5. After sucrose was completely dissolved, 5.0 mL of alternansucrase was added and the reaction was carried out at 55 °C for 22 h followed by deactivation treatment at 95 °C for 15 min. The reaction mixture was stored at 4 °C prior to further purification

#### 2.1.2. Removal of Citric Acid Buffer, Residual Sugars and Deactivated Residual Enzyme

Preliminary purification of the reaction mixture was carried out via a preparative chromatographic system, built and maintained in-house, to remove citric acid buffer, residual sugar, and residual enzymes. Using high-purity starting samples eliminates the need for cation and anion exchange chromatography. Thus, 500 mL of reaction mixture was passed through a jacketed glass column packed with 300 mL of SEPABEADS™ SP70 resin (Mitsubishi Chemical, Tokyo, Japan). The flow rate was about 3 mL/min delivered by a peristaltic pump and the temperature of the jacketed column was held at 50 °C by using a circulating water bath. The column was then washed with 500 mL of water at the same flow rate. This step removes citric acid buffer, residual sugars, deactivated residual enzyme, and other water-soluble impurities. The adsorbed steviol glycosides were eluted from the column by using 500 mL of 70% food grade ethanol (*v/v*) at 50 °C at the same flow rate and dried under vacuum at 70 °C overnight, or until completely dry. The purified glycosylated reb C is a white powder, and contains about 6 major multi-α-glycosylated reb C products and residual reb C (Figure 2, above). The most abundant peak has a molecular weight corresponding to the addition of one glucose unit to the reb C backbone (reb C+1G).

#### 2.1.3. Isolation and Purification of the reb C+1G Compound

The purified glycosylated reb C sample was prepared in 25% methanol at 1% (*w/v*). The compound reb C+1G was separated from the glycosylated reb C mixture by an Agilent (Santa Clara, CA, USA) semi-preparative chromatography system equipped with a Phenomenex (Torrance, CA, USA) Kinetix XB-C18 column (150 mm × 21.2 mm, 5 µm), using 0.1% formic acid in water (solvent A) and methanol (solvent B) as mobile phases at a flow rate of 4 mL/min. UV detection at both 210 nm and 254 nm was used. The fraction containing reb C+1G was dried at 70 °C under vacuum, and the end product was a white crystal, which was different than the free-flow white powder form of reb A. Diffusion-ordered spectroscopy (Figure S7) showed highly similar diffusion constants for all signals, indicating that the crystal is likely one molecule, and not a mixture.

#### 2.1.4. Liquid Chromatography-Ultra Violet/Mass Spectrometry Analysis

LC-UV/MS analysis was carried out on a Waters Acquity ultra-high performance liquid chromatographic system (Milford, MA, USA) equipped with a photodiode-array detector (PDA) and a single quadrupole mass spectrometer (Acquity SQ Detector) with an electrospray ionization source (ESI) operating in negative mode. An Agilent Poroshell EC-C18 column (150 mm × 4.6 mm, 2.7 µm) held at 50° C was used for the separation with 1 mM ammonium fluoride (NH4F) in water (solvent A) and acetonitrile (solvent B) as mobile phases. The flow rate was 0.6 mL/min with an elution gradient as follows: 0–2 min 75% A, 2–5 min 75–68% A, 5–14 min 68% A, 14–16 min 68–50% A, 16–18 min 50% A, 18–18.5 min 50–10% A, 18.5–22 min 10% A, 22–22.5 min 10–75% A, and 22.5–28 min 75% A. The injection volume was 5 µL. The mass acquisition window was from 50 to 2000 Da.

#### 2.1.5. MS Product Ion Scan

**Figure 4:**
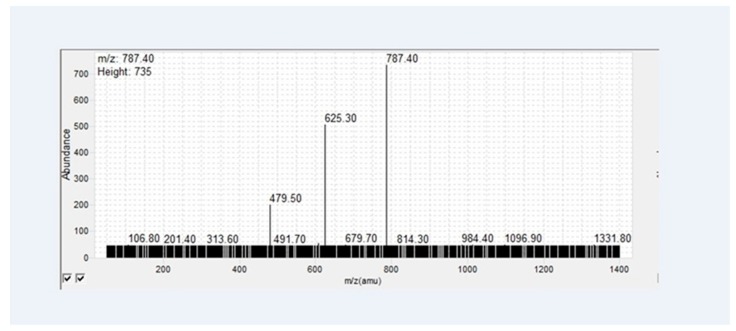
Product ion scan of reb C+1G.

The MS/MS product ion scan was carried out on an Agilent 6460 Triple Quadrupole MS, equipped with a Jet Stream ESI source operating in negative mode. Thus, 100 ppb of EMS sample in 30% acetonitrile was infused into the MS at a rate of 10 µL/min. The collision energy was ramped to find the one that produces the highest signal of the main product ion, *m/z* 784.4. The collision energy used for the mass spectrum shown in Figure 4 was 25 V.

#### 2.1.6. Nuclear Magnetic Resonance Spectroscopy (NMR)

Approximately 5 mg of reb C+1G was dissolved in 0.6 mL DMSO-*d*_6_ (Eurisotop, Saarbrücken, Germany) in a 5-mm NMR tube for spectral data acquisition. NMR spectra were recorded on two different NMR spectrometers: (1) an Avance III 600 MHz (Bruker, Karlsruhe, Germany) NMR spectrometer having a magnetic flux density of 14.1 Tesla and equipped with a BBO cryoprobe and an automated sample changer (Bruker B-ACS 120) and (2) an Avance III HD 500 MHz (Bruker, Karlsruhe, Germany) NMR spectrometer having a magnetic flux density of 11.7 Tesla and equipped with a BBO Prodigy cryoprobe and an automated sample changer (Bruker SampleXpress 60). All NMR spectra were recorded at 298 K and were referenced to internal tetramethylsilane (TMS, Sigma-Aldrich Chemie GmbH, Steinheim, Germany) at 0.0. ppm.

#### 2.1.7. Sensory Evaluation

Limited qualitative descriptive sensory evaluation of reb C+1G was performed by an internal panel. Aqueous solution sweetened with reb C or reb C+1G at 200 ppm (was tasted against a 2% sucrose reference solution. Relative sweetness (more, less, or same) of reb C or reb C+1G against the sucrose reference solution was determined the panel. The panel also evaluated the typical bitter or liquorice aftertaste associated with steviol glycoside. All samples were evaluated in duplicate.

## 3. Results and Discussion

### 3.1. Preliminary Structure Determination by Liquid Chromatography-Mass Spectrometry

**Figure 5:**
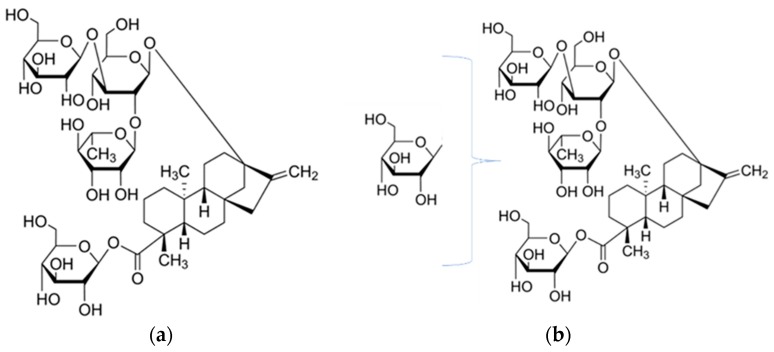
(**a**) Reb C; (**b**) reb C+1G (1 glucose (G) added to reb C backbone, position unknown).

Reb C is a steviol glycoside containing rhamnose (Figure 5). The mono-glucosyl rebaudioside C (reb C+1G) was isolated as a white crystalline material. It can be more easily handled than Reb A’s free flow white powder, which creates dust during application. The molecular weight of the compound was determined to be *m/z* 1111.5 by LC-MS analysis, corresponding to the [M − H]^−^ of a single glucose addition to reb C (Figure 5). Its main fragmentation pattern was further determined by an infusion MS/MS daughter ion scan (Figure 4). A recent publication reported that sugars linked at the C-19 (carboxylic group) side of a steviol glycoside are first cleaved at lower collision energies followed by stepwise fragmentation of the sugar moieties at the C-13 side [16]. The loss of two glucose units to form the main daughter ions of *m/z* 787.4 indicated that the glucose was added to the C-19 (carboxylic group) glucose of the reb C.

### 3.2. Nuclear Magnetic Resonance Structure Elucidation

**Figure 6:**
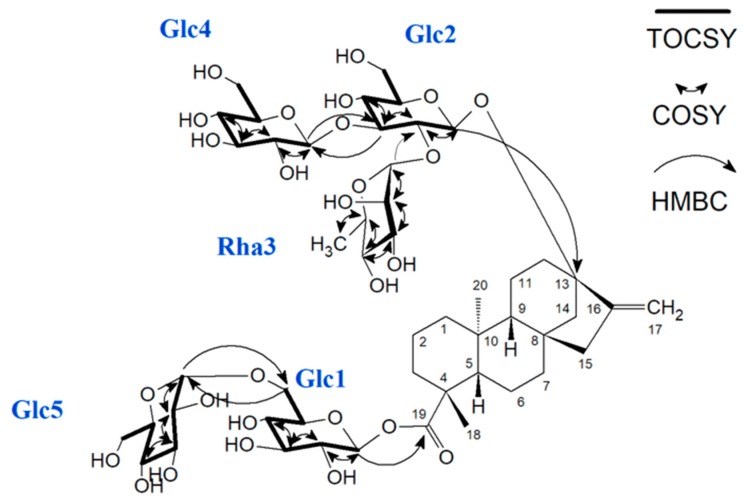
Structure of the α-1→6-glucosyl rebaudioside C with added glucose unit (1G) (reb C+1G). Arrows indicate key correlations used for structure elucidation. Glc5 is the added glucose moiety. TOCSY: ^1^H-^1^H total correlation spectroscopy; COSY: ^1^H-^1^H correlation spectroscopy; HMBC: ^1^H-^13^C heteronuclear multiple bond correlation.

A series of one- and two-dimensional NMR experiments, including ^1^H NMR, ^13^C NMR, ^1^H-^13^C heteronuclear single quantum coherence (HSQC), ^1^H-^13^C heteronuclear multiple bond correlation (HMBC), ^1^H-^1^H correlation spectroscopy (COSY), and ^1^H-^1^H total correlation spectroscopy (TOCSY) were performed to elucidate the structure of the product α-1→6-glucosyl rebaudioside C (reb C+1G) (Figure 6).

**Figure 7:**
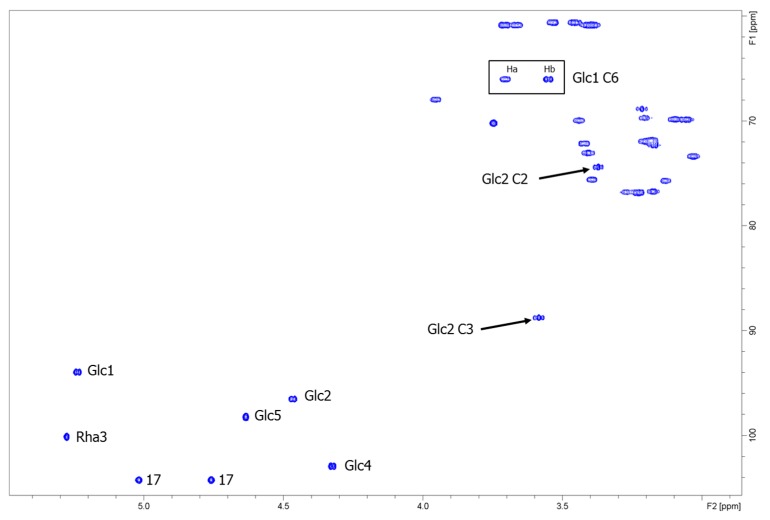
Carbohydrate region of the ^1^H-^13^C heteronuclear single quantum coherence (HSQC) spectrum of reb C+1G. Significant downfield shifts of Glc2 C2 and C3 (black arrows) and Glc1 C6 (black box) indicate linkage positions.

The ^1^H NMR spectrum of α-1→6-glucosyl rebaudioside C (reb C+1G, Figure S1) showed two methyl singlets at δ_H_ 1.13 (C-18) and δ_H_ 0.86 (C-20), two olefinic protons of an exocyclic double bond (C-17) as singlets at δ_H_ 4.76 and δ_H_ 5.02, and between δ_H_ 0.77-2.05 nine methylene (C-1,2,3,6,7,11,12,14,15) and two methine (C-5,9) protons; these signals are characteristic for the *ent*-kaurene diterpenoid aglycone (steviol). Due to overlap of the hydroxyl signals and the anomeric protons, the number of saccharide residues was determined from the HSQC spectrum (Figure 7). Five anomeric protons, with three saccharide residues in the β-configuration (*J*_1,2_ ~8 Hz) and two saccharide residues in the α-configuration (*J*_1,2_ ~1 Hz), were observed. Complete scalar coupling networks for each of the five saccharide residues were identified using COSY (Figure S6) and TOCSY experiments, allowing identification of three β-Glc residues (Glc1, Glc2 and Glc4), one α-Glc residue (Glc5), and one α-Rha residue (Rha3). Combining the information from both the TOCSY and the HSQC experiments allowed for complete assignment of the ^1^H and ^13^C chemical shifts for each saccharide residue (Table 1, Figures S3 and S4). Inter-residue connectivity was established using the HMBC data (Figure S5). Glc1 was identified by the observation of a long-range correlation between H-1 of Glc1 (δ_H_ 5.24) and C-19 (δ_C_ 175.7). Observation of a relatively downfield-shifted C-6 for Glc1 (Table 1) indicated that Glc1 was substituted at the 6-position of the saccharide. A long-range correlation was observed between H-1 of Glc5 (δ_H_ 4.64) and C-6 of Glc1 (δ_C_ 66.0), thus establishing the connectivity between the two saccharides bound to the steviol core via the C-19 carboxyl functionality. Glc2 was identified by a long-range correlation between H-1 of Glc2 (δ_H_ 4.46) and C-13 (δ_C_ 85.5). The ^13^C chemical shifts of Glc2 indicated that this saccharide was 2,3-branched (Table 1). The 2-substituent of Glc2 was identified by a long-range correlation between H1 of Rha3 (δ_H_ 5.28) and C-2 of Glc2 (δ_C_ 74.4), while the 3-substituent of Glc2 was identified by a long-range correlation between H-1 of Glc4 (δ_H_ 4.32) and C-3 of Glc2 (δ_C_ 88.7). A summary of key correlations used to elucidate the structure of α-1→6-glucosyl rebaudioside C (reb C+1G) is shown in Figure 6.

### 3.3. Sensory Evaluation

Sensory analysis revealed that reb C+1G was sweeter than 2% sucrose, corresponding to a sweetness potency of at least 100. Reb C was much less sweet than the reference, in line with a potency of 30 reported in literature [2]. In addition, reb C+1G had much less bitter or liquorice-type aftertaste than reb C. The increased sweetness and reduced bitterness resulted in noticeable improvement of organoleptic properties of the parent reb C sweetener.

## 4. Conclusions

A new enzymatic process was applied to modify high intensity sweetener rebaudioside C and a mono glycosylated reb C derivative was separated, purified, and characterized. NMR structure elucidation showed the additional glucose is added to the reb C backbone via a rare α-1→6 linkage. To our best knowledge, it is first time such a reb C derivative is reported. Due to the compound’s improved sensory and solubility property over its parent reb C, it has the potential to be commercialized as a low cost high intensity sweetener.

## Figures and Tables

**Table 1:** ^1^H and ^13^C NMR signal assignments for the α-1→6-glucosyl rebaudioside C+1G.

^1^H Chemical Shift δ (ppm)	^13^C Chemical Shift δ (ppm)	Multiplicity	Number H	Assignment
				**Steviol**
0.77, 1.78	40.0 *^a^*	multiplet	2	H-atoms at C-1
1.35, 1.79	18.6	multiplet	2	H-atoms at C-2
0.96, 2.05	37.4	multiplet	2	H-atoms at C-3
--	43.1	--	--	C-4
1.04	56.4	multiplet	1	H-atom at C-5
1.70, 1.98	21.1	multiplet	2	H-atoms at C-6
1.34, 1.48	41.0	multiplet	2	H-atoms at C-7
--	41.7	--	--	C-8
0.91	53.1	multiplet	1	H-atom at C-9
--	38.9 *^a^*	--	--	C-10
1.50, 1.68	20.0	multiplet	2	H-atoms at C-11
1.44, 1.84	36.7	multiplet	2	H-atoms at C-12
--	85.5	--	--	C-13
1.58, 2.02	42.3	multiplet	2	H-atoms at C-14
1.97, 2.07	47.5	multiplet	2	H-atoms at C-15
--	152.7	--	--	C-16
4.76, 5.02	104.3	multiplet	2	H-atoms at C-17
1.13	27.8	singlet	3	H-atoms at C-18
--	175.7	--	--	C-19
0.86	14.9	singlet	3	H-atoms at C-20
				**Glc1**
5.24	93.9	doublet, 8.2 Hz	1	H-atom at C-1
3.17 *^b^*	72.3 *^b^*		1	H-atom at C-2
3.27	76.8		1	H-atom at C-3
3.21	69.7		1	H-atom at C-4
3.40	75.6		1	H-atom at C-5
3.55, 3.70	66.0		2	H-atoms at C-6
				**Glc2**
4.46	96.5	doublet, 7.8 Hz	1	H-atom at C-1
3.37	74.4		1	H-atom at C-2
3.58	88.7		1	H-atom at C-3
3.21	68.8		1	H-atom at C-4
3.13	75.7		1	H-atom at C-5
3.40 *^c^*, 3.65	60.9 *^c^*		2	H-atoms at C-6
				**Rha3**
5.28	100.1	doublet, ~1 Hz	1	H-atom at C-1
3.75	70.2		1	H-atom at C-2
3.44	69.9		1	H-atom at C-3
3.18 *^b^*	72.2 *^b^*		1	H-atom at C-4
3.95	67.9		1	H-atom at C-5
1.07	18.1		3	H-atoms at C-6
				**Glc4**
4.32	102.9	doublet, 7.9 Hz	1	H-atom at C-1
3.03	73.5		1	H-atom at C-2
3.17	76.7		1	H-atom at C-3
3.05	69.9		1	H-atom at C-4
3.23	77.0		1	H-atom at C-5
3.40 *^c^*, 3.71	60.9 *^c^*		2	H-atoms at C-6
				**Glc5**
4.64	98.2	doublet, ~1 Hz	1	H-atom at C-1
3.18 *^b^*	71.9 *^b^*		1	H-atom at C-2
3.41	73.0		1	H-atom at C-3
3.10	69.9		1	H-atom at C-4
3.42	72.1		1	H-atom at C-5
3.46, 3.53	60.7		2	H-atoms at C-6

*^a^* signal(s) obscured by DMSO residual; *^b^*^–*c*^ Overlapping signals denoted by like letters, chemical shift values are interchangeable.

## Supplementary Materials

The following are available online at http://www.mdpi.com/2218-273X/9/1/27/s1, Figure S1: ^1^H-NMR spectrum of reb C+1G, Figure S2: ^13^C-NMR spectrum of reb C+1G, Figure S3: HMBC-NMR spectrum of reb C+1G, Figure S4: Superimposed HSQC- and HMBC-NMR spectra of reb C+1G, Figure S5: Superimposed HSQC- and HMBC-NMR spectra of reb C+1G, Figure S6: COSY-NMR spectrum of reb C+1G, Figure S7: ^1^H DOSY-NMR of reb C+1G.

## References

[1] https://www.foodnavigator-usa.com/Article/2018/10/01/US-retail-sales-of-stevia-sweeteners-rose-11.9-in-the-past-year-as-sales-of-artificial-sweeteners-continue-to-slide.

[2] Prakash I., Markosyan A., Bunders C. (2015). Development of Next Generation Stevia Sweetener: Rebaudioside M. Foods.

[3] Olsson K., Carlsen S., Semmler A., Simón E., Mikkelsen M.D., Møller B.L. (2016). Microbial production of next-generation stevia sweeteners. Microb. Cell Fact..

[4] Prakash I., Bunders C., Devkota K.P., Charan R.D., Ramirez C., Priedemann C., Markosyan A. (2014). Isolation and Characterization of a Novel Rebaudioside M Isomer from a Bioconversion Reaction of Rebaudioside A and NMR Comparison Studies of Rebaudioside M Isolated from *Stevia rebaudiana* Bertoni and *Stevia rebaudiana* Morita. Biomolecules.

[5] Adari B.R., Alavala S., George S.A., Meshram H.M., Tiwari A.K., Akella V.S., Sarma S. (2016). Synthesis of rebaudioside-A by enzymatic transglycosylation of stevioside present in the leaves of *Stevia rebaudiana* Bertoni. Food Chem..

[6] Kochikyan V.T., Markosyan A.A., Abelyan L.A. (2006). Combined enzymatic modification of stevioside and rebaudioside A. Appl. Biochem. Microbiol..

[7] Gerwig G.J., te Poele E.M., Dijkhuizen L., Kamerling J.P. (2016). Chapter One—Stevia Glycosides: Chemical and Enzymatic Modifications of Their Carbohydrate Moieties to Improve the Sweet-Tasting Quality. Adv. Carbohydr. Chem. Biochem..

[8] Tanaka O. (1997). Improvement of taste of natural sweeteners. Pure Appl. Chem..

[9] https://www.foodnavigator-usa.com/Article/2018/03/20/Cargill-launches-EverSweet-fermented-steviol-glycosides.

[10] Uhler B., Yang Z. (2018). Rebaudioside A and other unreported steviol glycoside isomers found in the sweet tea leaf. Phytochem. Lett..

[11] (2017). FDA GRAS Notice, GRN No. 662. http://www.fda.gov/Food/IngredientsPackagingLabeling/GRAS/NoticeInventory/default.htm.

[12] Cargill, Inc. (2008). FDA CFSAN/Office of food additive safety agency response letter to Cargill, Inc. regarding Notification of GRAS determination for sucromalt.

[13] Abelyan V.A., Balayan A.M., Ghochikyan V.T., Markosyan A.A. (2004). Transglycosylation of stevioside by cyclodextrin glucanotransferases of various groups of microorganisms. Appl. Biochem. Microbiol..

[14] Koyama E., Kitazawa K., Ohori Y., Izawa O., Kakegawa K., Fujino A., Ui M. (2003). In vitro metabolism of the glycosidic sweeteners, stevia mixture and enzymatically modified stevia in human intestinal microflora. Food Chem. Toxicol..

[15] Te Poele E.M., Devlamynck T., Jäger M., Gerwig J.G., van de Walle D., Dewettinck K., Hirsch K.H.A., Kamerling P.J., Soetaert W., Dijkhuizen L. (2018). Glucansucrase (mutant) enzymes from Lactobacillus reuteri 180 efficiently transglucosylate Stevia component rebaudioside A, resulting in a superior taste. Sci. Rep..

[16] Perera W.H., Avula B., Khan I.A., McChesney J. (2017). Assignment of sugar arrangement in branched steviol glycosides using electrospray ionization quadrupole time-of-flight tandem mass spectrometry. Rapid Commun. Mass Spectrom..

