# Supplementary material for: Enzymatic Synthesis and Characterization of a Novel α-1→6-Glucosyl Rebaudioside C Derivative Sweetener
Source: Biomolecules. 2019 Jan 14;9(1):27. doi: 10.3390/biom9010027 (PMC6358748; doi:10.3390/biom9010027)
Supplement: Supplementary file 1 [file biomolecules-09-00027-s001.pdf]

biomolecules

MDPI

Article

Enzymatic Synthesis and Characterization of a Novel α-1→6-Glucosyl Rebaudioside C Derivative Sweetener

Supplementary Materials:

Figure S1: 1H-NMR spectrum of reb C+1G. reb C: rebaudioside C. G: glucose

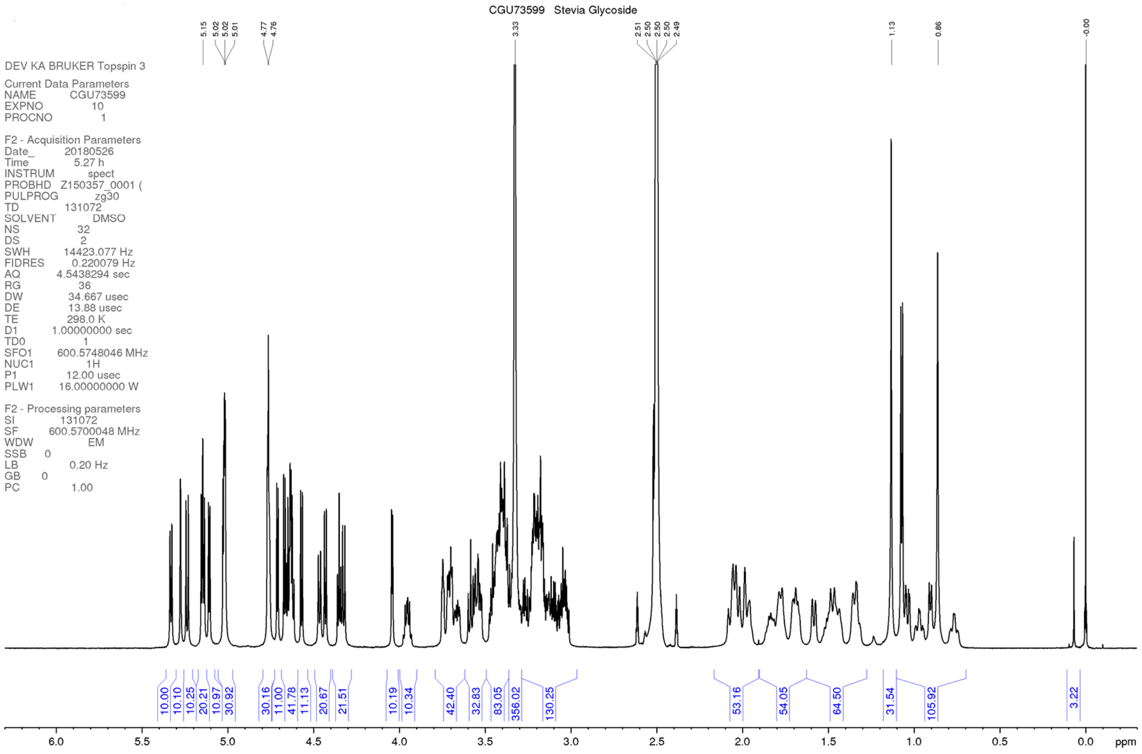

Figure S2: 13C-NMR spectrum of reb C+1G

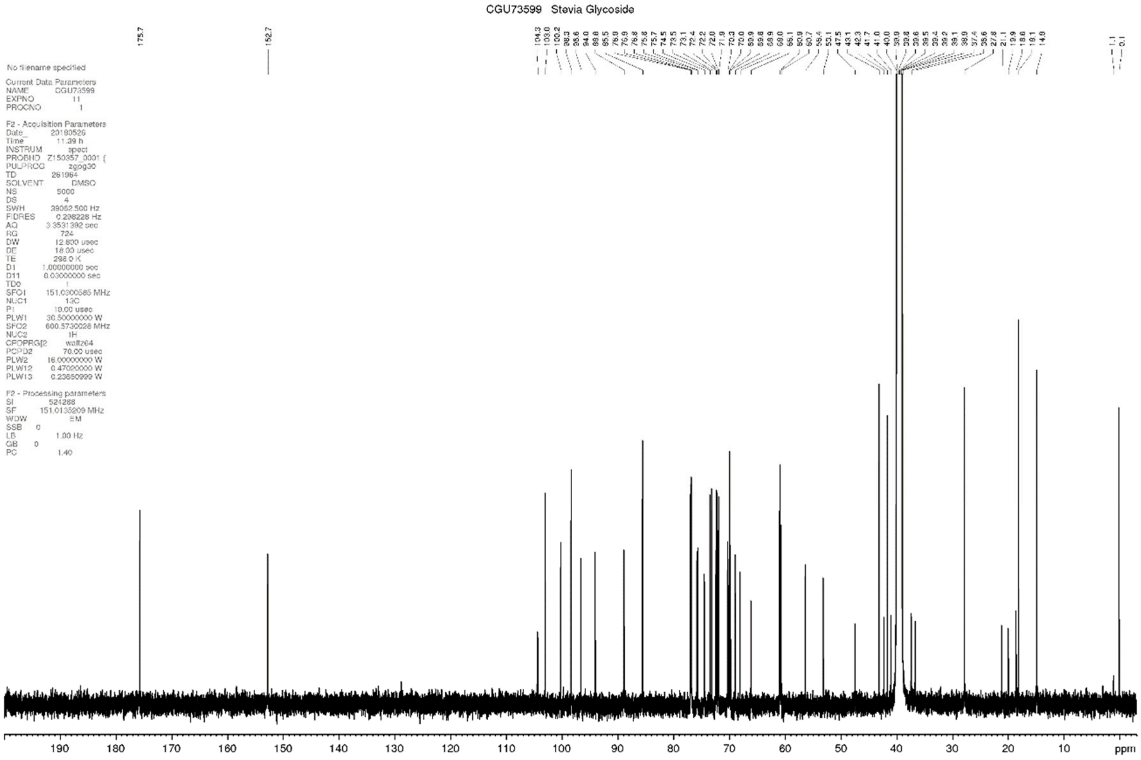

Figure S3: HMBC-NMR spectrum of of reb C+1G

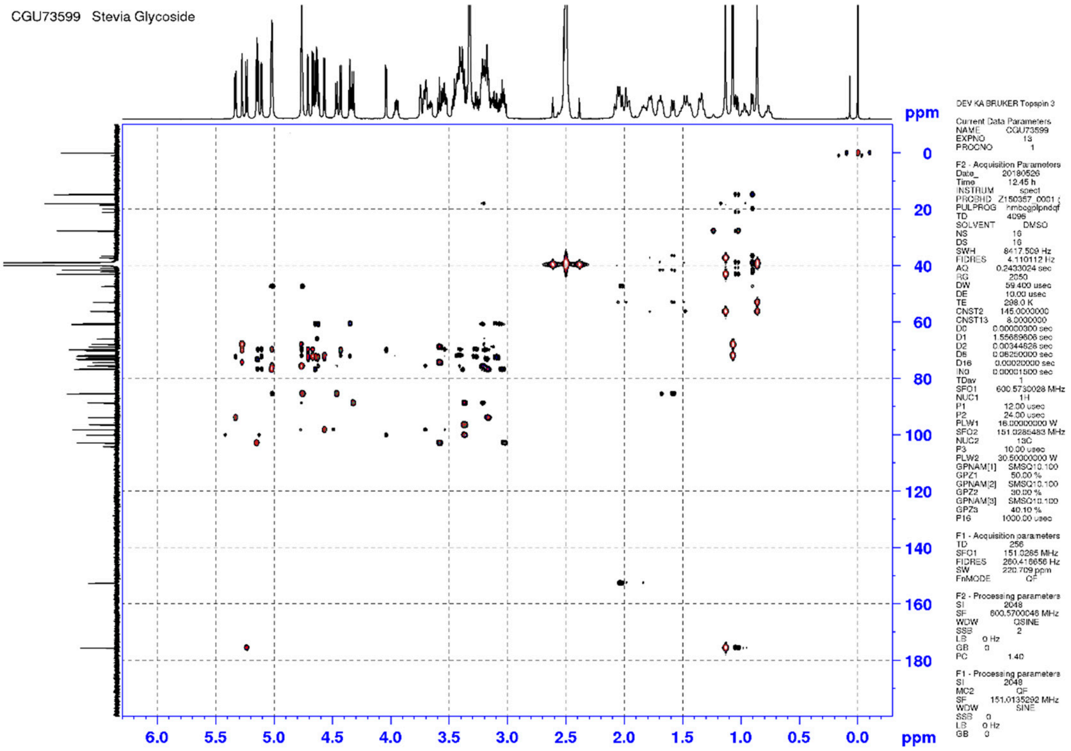

Figure S4: : Superimposed HSQC- and HMBC-NMR spectra of reb C+1G

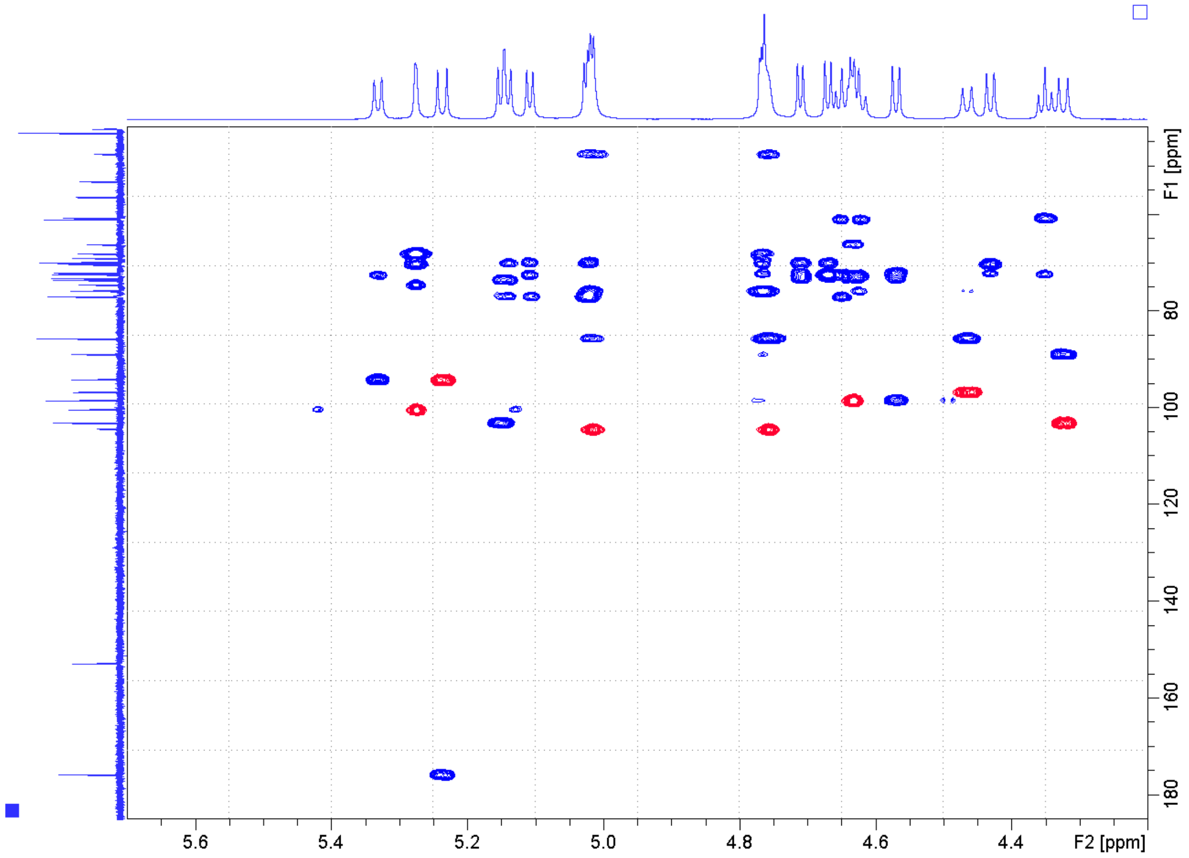

Figure S5: Superimposed HSQC- and HMBC-NMR spectra of reb C+1G

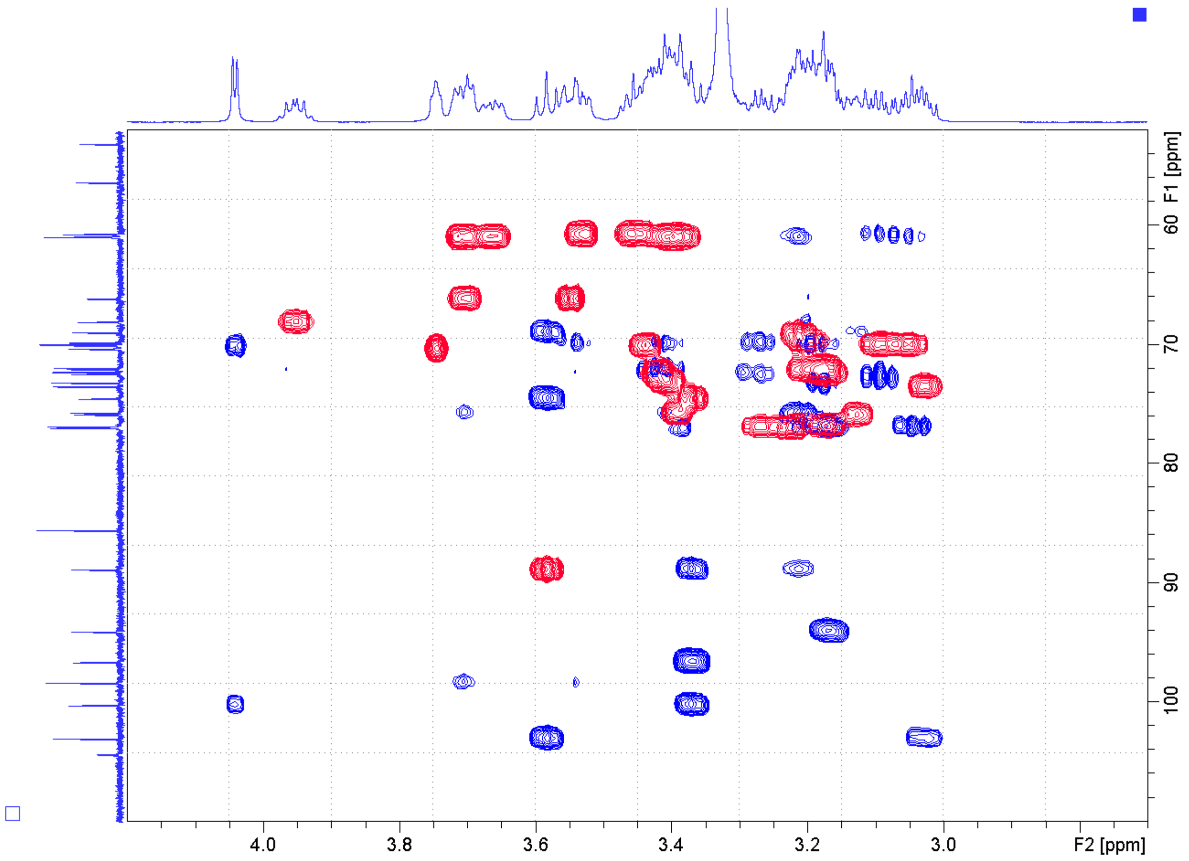

Figure S6: COSY-NMR spectrum of reb C+1G

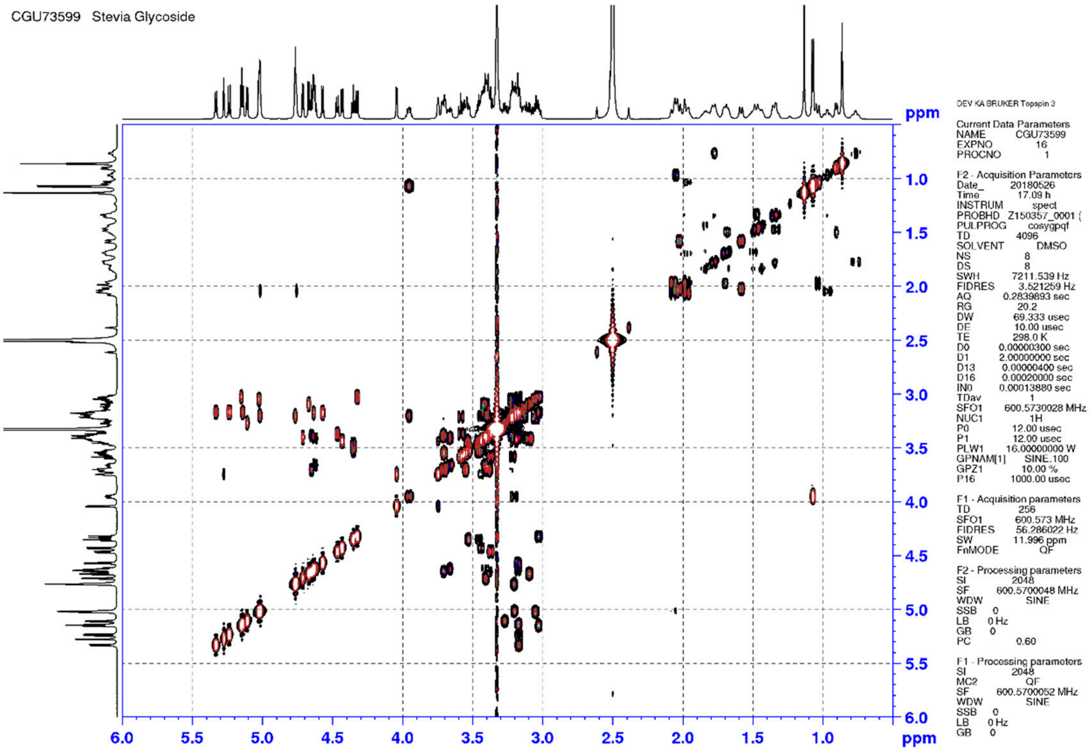

Figure S7: 1H DOSY-NMR of reb C+1G

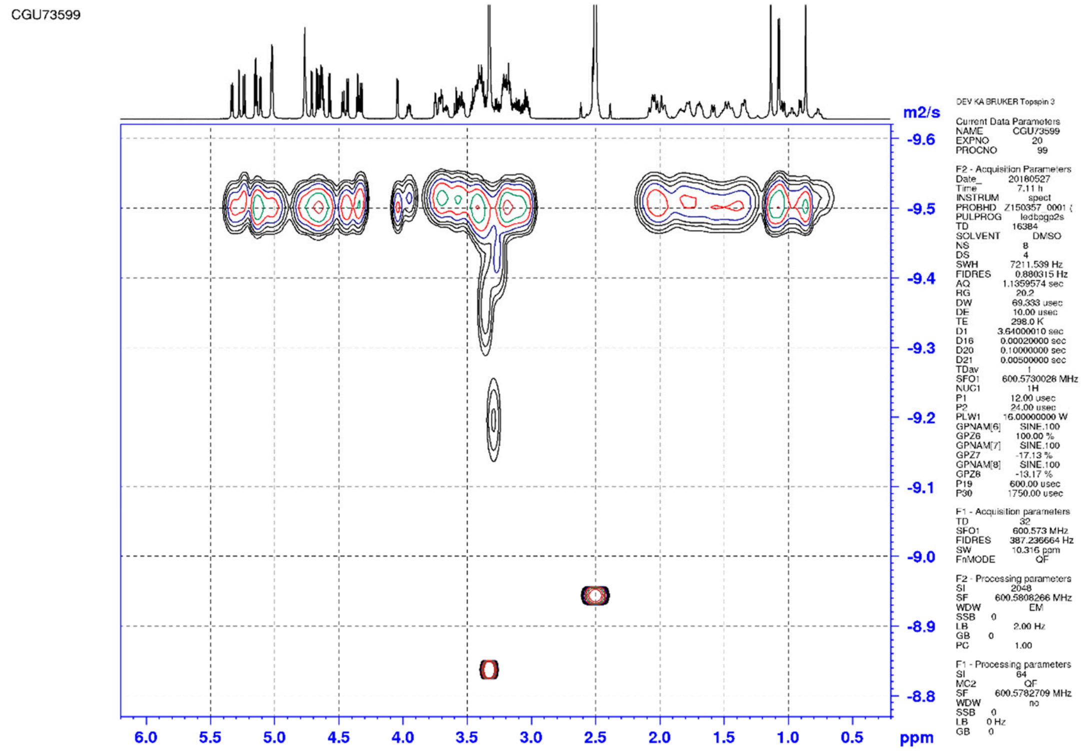

© 2018 by the authors. Submitted for possible open access publication under the terms and conditions of the Creative Commons Attribution (CC BY) license (http://creativecommons.org/licenses/by/4.0/).
